# Vitamin C activates pyruvate dehydrogenase (PDH) targeting the mitochondrial tricarboxylic acid (TCA) cycle in hypoxic *KRAS* mutant colon cancer

**DOI:** 10.7150/thno.51265

**Published:** 2021-01-25

**Authors:** Aiora Cenigaonandia-Campillo, Roberto Serna-Blasco, Laura Gómez-Ocabo, Sonia Solanes-Casado, Natalia Baños-Herraiz, Laura Del Puerto-Nevado, Jose Antonio Cañas, María Jesús Aceñero, Jesús García-Foncillas, Óscar Aguilera

**Affiliations:** 1 Translational Oncology Division, Oncohealth Institute, IIS-Fundacion Jimenez Diaz-UAM (Madrid), Spain.; 2 Hospital Puerta De Hierro Majadahonda, Majadahonda (Madrid), Spain.; 3 Department of immunology, IIS-Fundacion Jimenez Diaz-UAM (Madrid), Spain.; 4 Hospital General Universitario Gregorio Marañón. Universidad Complutense de Madrid. (Madrid), Spain.; 5 Universidad Católica San Antonio (Murcia), Spain.

**Keywords:** cancer, metabolism, PDK-1, vitamin C, KRAS, chemoresistance, hypoxia

## Abstract

**Background:** In hypoxic tumors, positive feedback between oncogenic KRAS and HIF-1α involves impressive metabolic changes correlating with drug resistance and poor prognosis in colorectal cancer. Up to date, designed KRAS-targeting molecules do not show clear benefits in patient overall survival (POS) so pharmacological modulation of aberrant tricarboxylic acid (TCA) cycle in hypoxic cancer has been proposed as a metabolic vulnerability of KRAS-driven tumors.

**Methods:** Annexin V-FITC and cell viability assays were carried out in order to verify vitamin C citotoxicity in KRAS mutant SW480 and DLD1 as well as in Immortalized Human Colonic Epithelial Cells (HCEC). HIF1a expression and activity were determined by western blot and functional analysis assays. HIF1a direct targets GLUT1 and PDK1 expression was checked using western blot and qRT-PCR. Inmunohistochemical assays were perfomed in tumors derived from murine xenografts in order to validate previous observations *in vivo*. Vitamin C dependent PDH expression and activity modulation were detected by western blot and colorimetric activity assays. Acetyl-Coa levels and citrate synthase activity were assessed using colorimetric/fluorometric activity assays. Mitochondrial membrane potential (Δ*ψ*) and cell ATP levels were assayed using fluorometric and luminescent test.

**Results:** PDK-1 in *KRAS* mutant CRC cells and murine xenografts was downregulated using pharmacological doses of vitamin C through the proline hydroxylation (Pro402) of the Hypoxia inducible factor-1(HIF-1)α, correlating with decreased expression of the glucose transporter 1 (GLUT-1) in both models. Vitamin C induced remarkable ATP depletion, rapid mitochondrial Δ*ψ* dissipation and diminished pyruvate dehydrogenase E1-α phosphorylation at Serine 293, then boosting PDH and citrate synthase activity.

**Conclusion:** We report a striking and previously non reported role of vitamin C in the regulation of the pyruvate dehydrogenase (PDH) activity, then modulating the TCA cycle and mitochondrial metabolism in *KRAS* mutant colon cancer. Potential impact of vitamin C in the clinical management of anti-EGFR chemoresistant colorectal neoplasias should be further considered.

## Introduction

Although epidermal growth factor receptor (EGFR) inhibitors have been employed to treat colorectal cancer (CRC) for decades with great success in patients with EGFR mutations, acquired-resistance inevitably occurs after long-term exposure, especially in those patients harboring mutations in *KRAS* gene.

Alterations in TCA cycle are often observed in association with HIF-1 overexpression and KRAS mutation in CRC 1. As it has been described, *KRAS* and *BRAF* mutations differentially regulate the hypoxic induction of HIF-1α and HIF-2α in colon cancer 2.

TCA cycle serves as a convergence point in the cellular respiration machinery, which integrates multiple fuel sources including glucose, glutamine, and fatty acids. Although both tumoral and non-tumoral cells can catabolize all principal types of substrates, they differ in the rate of uptake and catabolism of each fuel. While glucose provides the main source of pyruvate entering the TCA cycle in normal cells, cancer cells often shunt glucose away from the TCA cycle for catabolism through anaerobic glycolysis, and thus are more dependent on glutamine and fatty acids to reload TCA cycle intermediates.

Healthy normal cells rely on oxidative phosphorylation for ATP production. Glycolisis is the responsible for the oxidation of glucose into pyruvate to produce ATP. Pyruvate is then decarboxylated into acetyl-Coa as it enters into the mitochondria, allowing to boost the TCA cycle. Cancer cells however, depend on aerobic glycolysis for energy production, rewiring piruvate to lactate and downregulating catalytic TCA cycle.

Genetic alterations in Krebs cycle enzymes and/or aberrant expression of the TCA cycle drivers correlate with increased fatty acid β-oxidations and epithelial mesenchymal transition (EMT) induction 3, which are both characteristics of cancer pathogenesis. Alterations in the TCA cycle are, thus, of an increasingly attractive interest among the oncologists as potential prognostic markers in cancer and putative targets for the design of novel antitumor molecules.

Pyruvate dehydrogenase kinase-1 (PDK-1), a key mitochondrial enzyme overexpressed in many cancer cells, redirects glucose metabolism from oxidative phosphorylation toward aerobic glycolysis. The pyruvate dehydrogenase complex (PDC) decarboxylates pyruvate to acetyl CoA, which is further metabolized in the mitochondria 4. PDKs impair this process inactivating the PDC complex through phosphorylation of the E1α subunit.

Recently, aberrant overexpression of PDK-1 has been associated to poor prognosis and liver metastasis in colorectal cancer 5 and some reports show that liver-metastatic breast cancer cells also display high expression of PDK-1, strongly pointing out to a key role of this enzyme in liver metastasis independently of the origin of the primary tumor 6.

Interestingly, PDK-1 has been also described to promote tumor cell proliferation and migration in non-small cell lung cancer (NSCLC) by enhancing the Warburg effect 7 and previous works demonstrate tight correlation between abnormal cancer cell metabolism and *KRAS* mutation in several neoplasias. In 1931, the Nobel Prize in Medicine Otto Warburg stated that cancer was primarily caused by altered metabolism interfering with energy processing in the normal cell. Increased cell glycolytic rates even in the presence of oxygen is fully recognized as a hallmark in cancer and known as the Warburg effect.

The *KRAS* proto-oncogene encodes a ~21 kDa small GTPase, which cycles between GTP (guanosine-5'-triphosphate)-bound active and GDP (Guanosine 5′-diphosphate)-bound inactive states. Mutated *KRAS* is reported in approximately 35%-45% of colorectal cancers and >90% of pancreatic ductal adenocarcinoma (PDAC) correlating with abnormal metabolic rates, enhanced glucose uptake and resistance to EGFR-targeted therapies as well as preoperative chemoradiotherapy (CRT) 8-10.

As it has widely been described, several clinical trials have shown that *KRAS* mutations in cancer involve a lack of response to targeted anti-epidermal growth factor receptor (EGFR)-based therapies. Therefore, anti-EGFR therapies using cetuximab and/or panitumumab are now limited to patients with *KRAS* wild-type CRC 11-13.

Although several strategies to modulate *KRAS* aberrant activation have been designed, all attempts to target KRAS protein have failed in the clinical assays and it has been assumed to be invulnerable to chemotherapeutic attack.

It should be possible to design small molecules directly binding to GTP-binding site of KRAS then blocking its interaction with GTP. However, given the extraordinarily high affinity of KRAS for GTP and the abundance of GTP in cell cytoplasm (0.5 mmol/L) as well as the lack of well-defined hydrophobic pockets on the surface of RAS protein, this approach is currently considered unfeasible.

Evidences of the close link between mutant *KRAS*, the pyruvate dehydrogenase complex (PDHC) and the Warburg Effect in cancer are rapidly increasing.

In 2017, Trinidad AG *et al*, published that pyruvate dehydrogenase kinase 4, one of the 4 members of de PDK family, exhibited a novel role in the activation of mutant *KRAS*, regulating cell growth in lung and colorectal tumour cells. Moreover, depletion of PDHK4 caused disruption of KRAS cellular localization and therefore a reduction in KRAS activity which, in turn, resulted in reduced MAPK signalling 14.

Other works have reported that PDK inhibition of the pyruvate dehydrogenase complex is critical for the aberrant preferential activation of glycolysis, often observed in the Warburg metabolism 15.

In 1976, Linus Pauling and Ewan Cameron performed a pioneering clinical study of the survival times of 100 cancer patients who were given ascorbate (usually 10 g/day) plus adjuvant chemotheray and 1000 matched controls, patients who had received the same treatment except for the vitamin C. Survival times greater than 1 yr after the date of untreatability were observed for 22% of the ascorbate-treated patients and for 0.4% of the controls 16.

In 2015 an original article published in Science by Yun J *et al*., stated that oxidized vitamin C was able to kill CRC cells depending on the *KRAS* mutational status 17. They found that cultured human CRC cells harboring *KRAS* or *BRAF* mutations were selectively killed when exposed to high levels of vitamin C.

Later, in 2016 we published a scientific work partially confirming previous observations of Yun *et al*., but mainly focused on the putative interaction of vitamin C in the Warburg metabolism. In this work we described a novel antitumoral mechanism of vitamin C in *KRAS* mutant colorectal cancer involving the Warburg metabolic disruption through downregulation of key metabolic checkpoints in *KRAS* mutant cancer cells and tumors without killing human immortalized colonocytes 18.

More recently, it has also been described that Vitamin C is capable to kill hepatic cell lines and *BRAF* mutated thyroid cancer cells with high efficacy 19, 20.

Also, an interesting work carried out by Di Tano *et al*, (2020) pointed out to a synergistic effect of fasting-simulated diet and vitamin C against *KRAS* mutated cancers suggesting that vitamin C may represent a promising low toxicity intervention to be tested in randomized clinical trials against colorectal cancer 21.

We propose that pharmacological targeting of tricarboxylic acid (TCA) cycle in hypoxic and hard-to-treat neoplasias may constitute a novel approach in the treatment and/or chemosensibilization of KRAS driven tumors. Data here displayed places vitamin C as an unexpected capital inhibitor of tumor metabolism in *KRAS* mutant CRC, regulating the expression of PDK-1 and, therefore, the activity of the PDHC complex, boosting the conversion of pyruvate into Acetyl CoA within the mitochondria.

## Methods

### Cell culture and cell lines

SW480 and DLD1 cells were cultured in DMEM supplemented with 10% Fetal Bovine Serum (FBS) (Invitrogen) in a humidified atmosphere containing 5% CO2 at 37 °C. Cells lines were originally obtained from the American Type Culture Collection (ATCC) and authenticated using GenePrint® 10 system (Promega), which allows co-amplification and three-color detection of ten human loci: TH01, TPOX, WA, Amelogenin, CSF1PO, D16S539, D7S820, D13S317 and D5S818. Short Tandem Repeats profiles were sent for comparison against cell line databases (ATCC, DSMZ). Last test was done on February 2015.

SW480 derive from primary tumour and lymph-node metastasis and harbors a mutation in codon 12 of the RAS proto-oncogene (G12V).

DLD1 derive from primary tumor and carries the heterozygous *KRAS* G13D mutation.

Both cell lines neither were exposed to previous chemotherapy nor anti-EGFR treatment. Inmortalized human colonocytes (HCEC) were provided by Prof. Manel Esteller IDIBELL (Barcelona, Spain).

To mimic hypoxia conditions, hypoxia agent NiCl2 was added to the medium in the final concentration of 500 µM. MG-132, resuspended on DMSO, was used in order to block protein degradation by proteasome, it was added to the medium in the final concentration of 25 µM (up to a maximum of 6 hours) Vitamin C solution stock was prepared from L-Ascorbic acid powder diluted in Ringer-Lactate Buffer. L-Ascorbic acid, Nickel Chloride (NiCl2) were purchased from Sigma. MG-132 (462482 MG-132 5MG) was obtained from ENZO (Palex Medical, S.A).

### Antibodies

HIF-1α antibody 1:1000 (abcam reference #ab51608), GLUT1 antibody 1:1000 (Merk Millipore reference #07-1401), PDK1 antibody 1:1000 (abcam reference #ab110025), HIF-1α OH-P402 1:1000 (abcam reference #ab72775), Beta-actin antibody 1:10000 (cell signaling, Cat. #4967), Pyruvate dehydrogenase E1-alpha subunit 1:2000 (abcam reference #ab ab110334) and Pyruvate dehydrogenase E1-alpha subunit (phospho S293) 1:1000 (abcam reference #ab177461) were used for western blot. GLUT1 and PDK1 were used for Immunohistochemistry assays.

### Cell viability assays

HCEC, SW480 and DLD1 were treated with pharmacological concentrations of vitamin C (0-5 mM) for 24 hour. Then, cells were trypsinized and fixed with trypan blue solution (Sigma-Aldrich). Cell counting was carried out using a TC20TM Automatic Cell Counter (Biorad). For time-course treatment assay cell lines were treated with 5 mM vitamin C during 2, 4, 6, 8, 12 and 24 hours, after treatment cells were washed twice with PBS and then cells were cultured in DMEM supplemented with 10%. Survival percentage of cells was determinated with Cell Counting Kit assay (CCK8, Sigma Aldrich #96992).

### Apoptosis analysis

HCEC, SW480 and DLD1 were treated with vitamin C (5mM) for 24 hours. 3-5 × 10^5^ cells were collected by trypsinizing, centrifugation and resuspended in 100 μL of PBS. Afterwards, 5 μL of annexin V-FITC and 5 μL of propidium iodide were added and incubated for 15 min in the dark, then 400 μL of PBS were added and samples were incubated for 45 min at RT in the dark. V-FITC binding was analyzed by flow cytometry using a BD FACS Canto II device (Becton, Dickinson and Company) (Ex = 488 nm; Em = 350 nm) using FITC signal detector and PI staining by the phycoerythrin emission signal detector.

### Western blots

Protein samples (25 μg protein/lane) were resolved by 12% SDS-PAGE and electrotransferred onto transfer membranes that were blocked with 5% Skim milk () for 1 hour at RT. The blots were incubated overnight at 4oC with antibodies of interest (mentioned before). After washing four times with TBS-T (0.1% Tween-20 in Tris-buffered saline, TBS) the blots were incubated with horseradish peroxidase-labeled goat anti-mouse or anti-rabbit IgG antibodies, then membranes were washed again four times with TBS-T. Inmunoblots were visualized using an enhancer chemiluminiscence detection kit (ECL Prime; GE Healthcare) and imaged using an Amershan Imager 600 All western blot images are representative of 3 different experiments or biological repeats.

### HIF 1α functional analysis

In order to mimic hypoxia conditions, cells were pretreated with NiCl2, ascorbate or both along 12 hours for SW480 and during 24 hours for DLD1. In previous HIF1α measurements, the nuclei were isolated using a Nuclear Extraction Kit (abcam). The total amount of HIF1α in cell lysates was determined by HIF1α transcription factor assay Kit from abcam (ab133104) per manufacturer instructions.

### Mitochondrial membrane potential (*ΔΨ*m) measurements

DLD-1 and SW480 cells were cultivated in P96-well plate and treated with NiCl2 (500µM), ascorbate or both for 2, 4 and 6 hours. *ΔΨ*m determination was carried out using an JC-1 - Mitochondrial Membrane Potential Assay Kit (abcam).

### ATP measurements

DLD-1 and SW480 cells were cultivated in P96-well plate and treated with NiCl2 (500 µM), ascorbate or both for 2 and 6 hours. ATP determination was carried out using an ATP Luminiscent assay from abcam (abcam).

### RT-PCRq

Total RNA was isolated from SW480 and DLD1 cells using an RNeasy Mini Kit (Qiagen) following the manufacturers protocol. mRNA quality and quantity was spectrophotometrically assessed using a NanoDrop 200 UV/Vis spectrophotometer. cDNA was synthesized from total mRNA using the Advantage RT-for-PCR kit (Clonetech Laboratories) following the manufacturers protocol. PCR analysis of target sequences were generated using the Advantage cDNA kit (Clonetech Laboratories) with the following PCR primers; GLUT1: 50 - TCATCAACCGCAACGAGGAG - 30(Forward), 50 - CAAAGATGGC- CACGATGCTC - 30 (Reverse); BNIP3: 50 - TGGACGGAGTAGCTC- CAAGA - 30 (Forward), 50 - TCATGACGCTCGTGTTCCTC - 30 (Reverse); bactin: 50 - GCTGCTCGTCGACAACGGCTC - 30 (Forward), 50 - CAAACATGATCTGGGTCATCTTCTC - 30 (Reverse). PCR condition: 95 _C 1 min; 25 cycles of 95 _C 30 s, 57 _C 1 min, 72 _C 2 min; 72 _C 5 min. PCR products were separated on 1% agarose gel containing GelRedTM nucleic acid stain (Biotium) and visualized by UV using a PhotoDyne Imaging system (PhotoDyne Technologies). cDNA and PCR products were generated using MJ Mini Personal Thermal Cycler (Biorad).

### Immunohistochemistry assays

The paraffin embedded sections were cleared and the sections were incubated with 0.1% Pronase (Roche #165 921) in 0.1% CaCl2 pH 7.8. at 37C for 10 minutes. They were blocked with 3% H2O2 in TBS for 10 mins., washed then blocked with Dako Biotin Blocking System (Dako X0590). After washing, they were further blocked with 10% Normal Rabbit Serum for 10 mins at room temperature (RT) and incubated firstly with PDK1 (Abcam, ab110025) dilution 1:50, and GLUT-1 (Abcam, ab652) dilution 1: 1000 for 1h at RT, then with biotinylated Rabbit Anti-Mouse (Dako, E-0354) at 1/100 for 30 mins. at RT, and finally with Strep-ABC complex (Dako, K-0377) at 1/100 for 30 mins. at RT. The sections were developed with AEC substrate kit (vector lab, SK-4200) at RT for 20 mins., counterstained with haematoxylin and mounted with DAKO aqueous mount (Dako, 003181).

### Measurements of PDH, citrate synthase activity and acetyl-CoA levels

PDH activity was determined using the PDH Activity Colorimetric Assay Kit (Biovision, Mountain View, CA) according to the manufacturer's instructions. Briefly 3×106 cells were seeded and pretreated with NiCl2 (500 μM), ascorbate or combination along 8 hours for SW480 and during 24h for DLD1. Cells were lysed in PDH buffer and PDH activity was measured by the kinetic reduction of NAD to NADH, which resulted in a colorimetric (450 nm) product proportional to the enzymatic activity present. Citrate synthase activity was quantified using a Citrate Synthase (CS) Activity Colorimetric Assay Kit (BioVision, Milpitas, CA), according to the manufacturers' instructions at the same conditions. Acetyl-CoA levels were measured by Pico-Probe Acetyl-CoA Fluorometric Assay kit (BioVision, Milpitas, CA) according to manufacturer's instructions. All assays were normalized with protein concentrations.

### Murine assays

SW480 *KRAS* (G12V) cells were used to generate xenograft model in female athymic nude mice Foxn1nu, 7 weeks old (Harlan Laboratories). Suspensions of 2×10^6^ cells were injected subcutaneously in control and experimental mice. Once the tumor size volume reached 100 mm^3^, the mice were treated with intraperitoneal (i.p) vehicle or vitamin C (4gr/Kg) once daily with the same dosing schedule (SW480: n = 10) for 15 days. Tumors were measured twice weekly in three perpendicular dimensions using a vernier caliper and tumor volume was calculated using the ellipsoidal formula V (mm^3^) = 1/6 π × length (mm) × width^2^ (mm^2^).

### Statistical analysis

Data are expressed as the mean ± S.E. Differences in measured variables were assessed with ANOVA test and p value of <0.05 was considered significant. Statistical evaluation was performed using SPSS 18.0.2 and GraphPad Prism 6.

## Results and Discussion

### Vitamin C selectively kills KRAS mutant CRC cell lines without affecting normal colon cells

We aimed to investigate whether vitamin C could have some antitumoral activity in chemoresistant CRC in assays carried out using SW480 and DLD1 cell lines, displaying *KRAS* mutations and no sensitivity to cetuximab.

**Figure 1:**
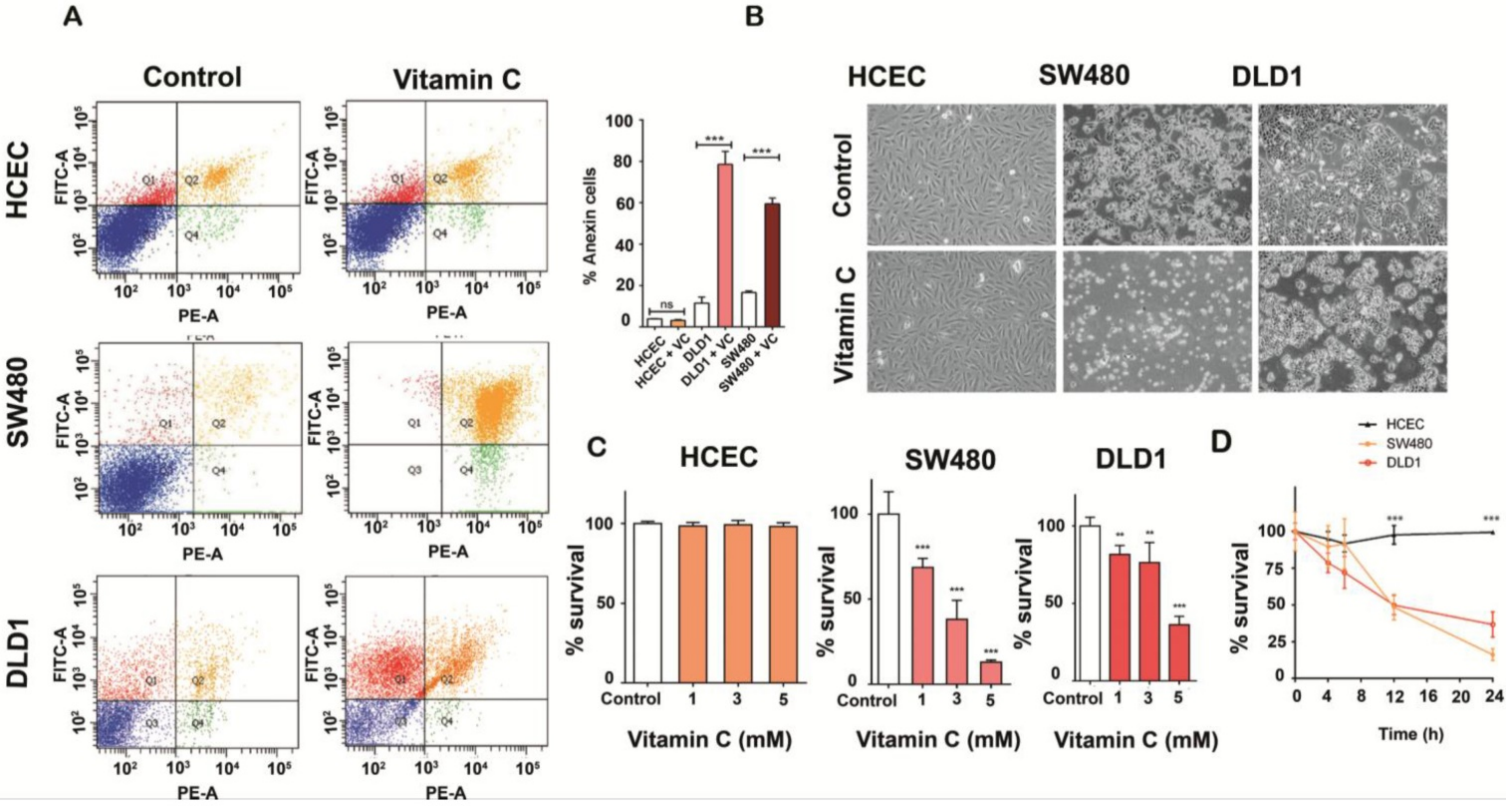
** Vitamin C selectively kills KRAS mutant colon cancer cells A.** Apoptosis-induced by vitamin C in normal human immortalized Colonocytes (HCEC), SW480 and DLD1 cancer cell lines were annalyzed by Annexin-PI assay. Each cell line (2,5 105 cells) was incubated with vitamin C (10 mM) and PBS for controls for 24 h. Apoptosis in each cell line was measured by staining with FITC-conjugated Annexin-V and Propidium Iodide (PI) using a Sigma-Aldrich Apoptosis kit. The populations of cells (annexin-V positive/PI negative) and late apoptotic cells (annexin-V positive/PI positive) as a percent of total cells were evaluated. Vitamin C displayed a selective killing effect on SW480 and DLD1. One-way ANOVA followed by Dunnett's post-test for multiple comparisons. *p< 0.05, **p < 0.001, n = 3. **B**.images show apoptotic activity in KRAS mutant SW480 and DLD1 cell lines after vitamin C treatment (10 mM) for 24 h. **C.** vitamin C treatment at different concentrations were carried out with HCEC and DLD1 and SW480 cancer lines. All cell lines were exposed to ascorbate at 1, 3 and 5mM, for 24 h. Then, cells were tripsinized and fixed with trypan blue solution (Sigma-Aldrich). Cell counting was carried out using a neubauer chamber (SigmaAldrich) One-way ANOVA followed by Dunnett's posttest for multiple comparisons < 0.05, **p < 0.001, n = 3. **D**. HCEC, SW480 and DLD1 cells were treated with 5Mm and 6Mm vitamin C respectively for 4,6, 12 and 24 h. Then, cells were tripsinized and fixed with trypan blue solution (SigmaAldrich). Cell counting was carried out using a neubauer chamber (SigmaAldrich) One-way ANOVA followed by Dunnett's posttest for multiple comparisons. *p < 0.05, **p < 0.001, n = 3.

SW480 and DLD1 cancer cells both harboring *KRAS* mutation (G12V and G13D respectively) and immortalized human colonocytes (HCEC) (*KRAS* wild type) were exposed to ascorbate for 2h to simulate clinical pharmacokinetics, and the effective concentration that decreased survival to 50% (EC50) was determined. Observed EC50 was ≤ 10 mM for both tumor cells lines tested. Remarkably, significative cytotoxicity in HCEC cells treated with 5 mM ascorbate was not detected (Figure 1A-1B). Mortality observed for both cancer cell lines was ≥ 60% just 36 h. after treatment (Figure 1C), although DLD1 displayed more resistance to ascorbate. We conclude that the effect of vitamin C seems to be, at least in part, dependent on the mutational status of KRAS as previously stated by Yun J *et al*. 17. Interestingly, KRAS mutation has been demonstrated to drive aberrant changes in the cell metabolic homeostasis in colon and pancreatic tumors 22, 23.

### HIF-1α stability and transcriptional activity are both inhibited by vitamin C in KRAS mutant CRC

As it has been previously reported, the hypoxic microenvironment of cancer cells results in drug resistance. Hypoxia associates with many signaling pathways, including PI3K/AKT, MAPK, or NOTCH and, interestingly, HIF-1 α protein expression is higher in cells displaying *KRAS* mutation 2.

Hypoxia-inducible factor 1 (HIF-1) is a heterodimeric protein that consists of two subunits, HIF-1α and HIF-1β. HIF-1 activates the transcription of many genes encoding for proteins that are involved in glucose metabolism, cancer proliferation, angiogenesis, metastasis and resistance to apoptosis 24.

High expression of HIF-1 has been detected in a vast number of neoplasias boosting the Warburg effect, and aberrant expression has been associated to poor prognosis in human colon cancer 25, 26.

**Figure 2:**
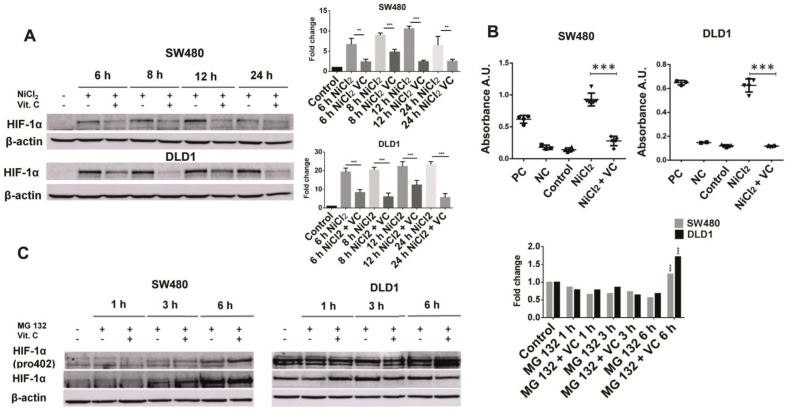
** Vitamin C reduces HIF1a activity and stability. A.** HIF1a expression in *KRAS* mutant cell lines SW480 and DLD1 treated with vitamin C (5 Mm; 6 mM) and NiCl2 (0.5 Mm) for 6, 8, 12 and 24 h. NiCl2 upregulates HIF1a expression mimicking tumor conditions which is downreguated when vitamin C is added. **B.** HIF1a activity in *KRAS* mutant cell lines SW480 and DLD1 treated with vitamin C (5 Mm; 6 mM) and NiCl2 (0.5 Mm). Vitamin C is able to downregulate the HIF1 increased transcriptional activity showed when cells are treated with Nicl2. **C.** Hidroxilated HIF1a expression in *KRAS* mutant cell lines SW480 and DLD1 treated with vitamin C (5 mM; 6 mM) and MG132 for 1, 3 and 6 h. Vitamin C induces hidroxilated HIF1a accumulation when proteasome is blocked at 6h.

As we can see (Figure 2A), exposure to 500 μM NiCl_2_ led to an increased and sustained stabilization of HIF1α subunit in SW480 and DLD1 cancer cells, an effect that vitamin C was able to reverse. In fact, after 6 and 8 h of exposure to ascorbate, HIF-1α was hardly detected in both SW480 and DLD1. In control assays displayed in Figure S1 we checked out that NiCl2 exposure neither affected cell viability nor induced apoptosis is a significant manner.

We also performed assays to check out if HIF-1 transcriptional activity might also be affected by vitamin C. In this experiment, a specific double stranded DNA (dsDNA) sequence containing the HIF-1α response element (5'-ACGTG-3') is immobilized inside of a 96-well plate. HIF-1α present in the cell nuclear extract, binds specifically to the HIF-1α response element. The HIF transcription factor complex is then detected by addition of a specific primary antibody directed against HIF1α and a secondary antibody conjugated to HRP is consequently added to provide a sensitive colorimetric reaction.Both cell lines were treated with 500 μM NiCl_2_ and 5mM vitamin C for 6 h.

As it is displayed in Figure 2B, addition of NiCl_2_ led to a huge increase of HIF-1α binding to the above mentioned HIF-1α response element. Vitamin C treatment led to a massive inhibition of HIF1α transcriptional activity, similar to that observed for the negative control provided in the assay.

Previous works have reported that the Factor Inhibiting HIF-1 (FIH-1) is involved in the cellular response to hypoxia physically interacting with HIF-1, blocking its transcriptional activity under normoxic conditions 27. The hypothesis that the inhibition of the transcriptional activity of HIF1 could be mediated by a vitamin C-dependent increase of FIH1 expression does not sound too far-fetched.

HIF-1α possesses an oxygen dependent degradation domain, containing two prolines in positions 402 and 564 that are hydroxylated in presence high O_2_ pressure. After hydroxylation, HIF1α binds to the pVHL-ubiquitin ligase complex and it is directed to proteasome for degradation 28. NiCl_2_ is added to the cell cultures to mimic hypoxia conditions and avoiding hydroxylation of both prolines. We wondered whether vitamin C might exert its activity on HIF-1α reverting NiCl_2_ hydroxylation protective effect.

For this purpose, we treated both cell lines with 25 μM of MG-132 (a proteasome inhibitor) up to a maximum of 6 hours. Again, 500 μM NiCl_2_ was also added to simulate hypoxia conditions. Then, cells were treated with 5 mM ascorbate for 1, 3 and 6 h.

In SW480 cells, a time dependent accumulation of HIF-1α was observed. Remarkably, vitamin C induced an observable accumulation of HIF-1 α (hydroxy P402) after 6 h of treatment (Figure 2C). So, Vitamin C leads to an increased proteasome degradation of HIF-1 α in cancer cells. Once again control assays were performed in order to ensure that MG132 did not affect cell viability (Figure S2).

Curiously, the effects observed in DLD1 were slightly different. HIF-1α did not experiment any kind of alteration, and it remained equal at 1, 3 and 6 h. However, hydroxylated HIF-1 α (P402) displayed a massive increase after 6 h treatment with vitamin C.

In spite of the observed differences in HIF-1α accumulation, we can conclude that vitamin C is somehow able to counteract the protective effect of NiCl_2_ on HIF-1 α at proline 402.

### Vitamin C induces PDK-1 inhibition leading to increased mitochondrial PDH activity

HIF-1-dependent expression of pyruvate dehydrogenase kinase is a metabolic switch which is needed for cellular adaptation to hypoxia 29.

As it has been previously described, overexpression of PDK-1 in CRC is associated to poor prognosis and liver metastasis 4. Moreover, Chin *et al.,* reported that PDK1 silencing in SW480 and HCT116 CRC cells tightly correlates with both decreased viability and proliferation 5 enhancing the role of PDK1 in *KRAS* mutant neoplasias.

High expression of PDK-1 leads to enhanced phosphorylation of the pyruvate dehydrogenase complex and reduced TCA activity. Since we have previously reported that vitamin C uncouples the Warburg effect 18, we performed assays to check out whether expression and/or activity of PDK-1 might be affected by vitamin C.

Western blot analysis was performed using both DLD1 and SW480 *KRAS* mutant cancer cell lines. As described, in order to mimic hypoxic conditions cells were treated with 500μM NiCl_2_ in order to stabilize HIF-1 and enhance the Warburg effect 30.

**Figure 3:**
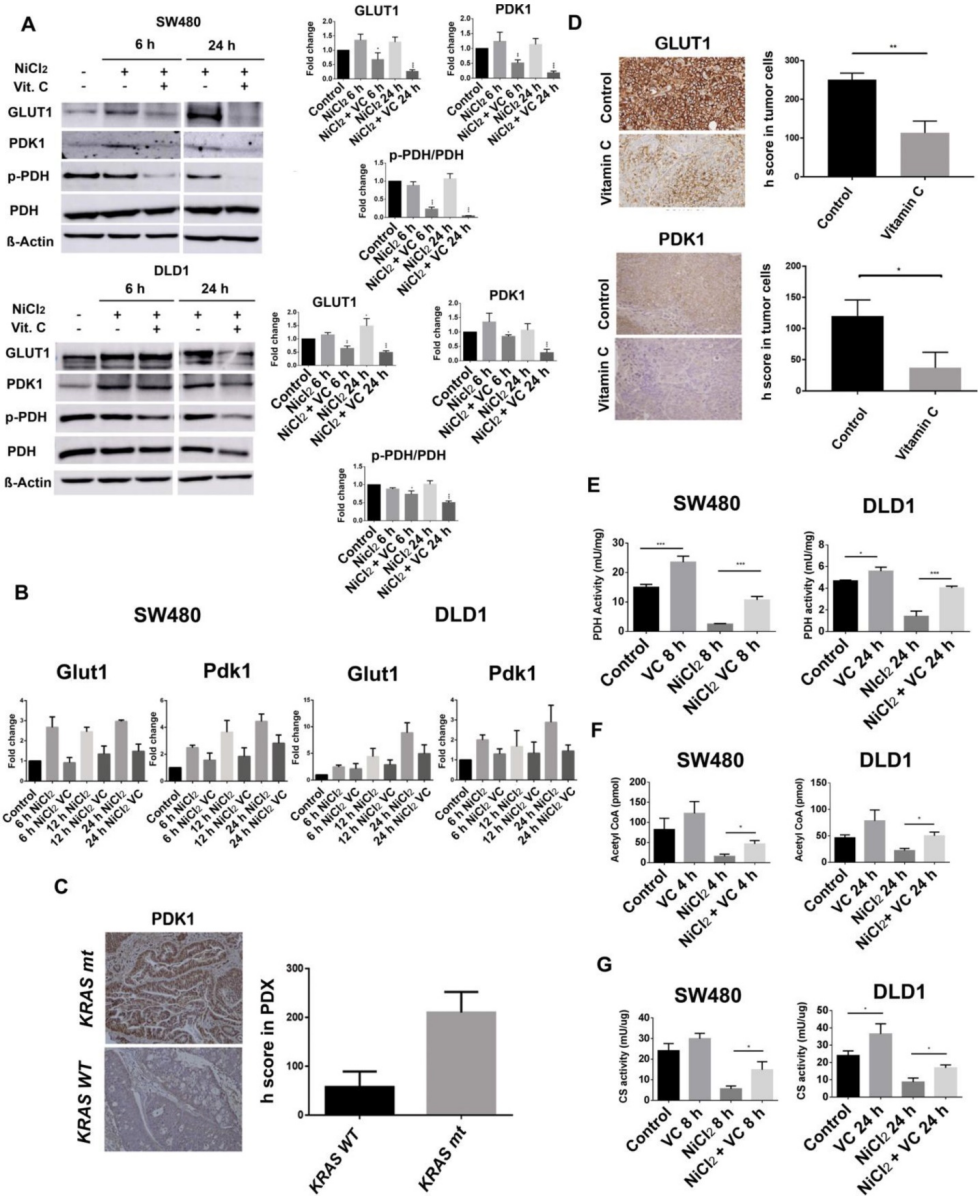
** Vitamin C interferes with PDK1-mediated phosphorylation of PDH. A** GLUT1, PDK1, p-PDH (S293) and PDH expression in KRAS mutant SW480 and DLD1 cell lines treated with vitamin C (5 Mm; 6 mM) and NiCl2 (0.5 Mm) for 6 and 24 h. Beta-actin was employed as protein loading control. NiCL2 upregulates PDK1, GLUT1 expression simulating hypoxic tumor conditions while vitamin C treatment reduces GLUT1, PDK1 and p-PDH expression** B.** qPCR analysis of Glut1 and Pdk1 in KRAS mutant SW480 and DLD1 cell lines treated with vitamin C (5 Mm; 6 mM) and NiCl2 (0.5 mM) for 6 and 24 h. Vitamin C downregulates the expression of Glut1 and Pdk1 at mRNA level**. C.**immunohistochemistry assays in colon cancer patient derived xenografts (PDX) show increased PDK1 expression in Kras mutant patients, n=3.** D.** immunohistochemistry assays in SW480 derived tumors shows *in vivo* downregulation of GLUT1 and PDK1 in vitamin C-treated tumors. Suspensions of 2×106 cells were injected subcutaneously in control and experimental mice Animals were treated (i.p) with vehicle or vitamin C (4 gr/kg body weight) for 15 days once daily. Umpaired t-test with Welchs correction *p < 0.05, **p < 0.001, n = 3.** E.** PDH activity in colon cancer SW480 and DLD1 cell lines treated with vitamin C (5 mM; 6 mM) alone or in combination with NiCl2(0.5 mM). Vitamin C rescues the inactivation produced by NiCl2 treatment and is able to activate PDH activity when used alone. One-way ANOVA followed by Dunnett's posttest for multiple comparisons. *p < 0.05, ***p < 0.001, n = 3.** F.** Acetyl-CoA detection in KRAS mutant SW480 and DLD1 cell lines when treated with NiCl2 (0.5 Mm) alone or in combination with vitamin C (5 mM; 6 mM). Vitamin C induces acetyl-CoA accumulation One-way ANOVA followed by Dunnett's posttest for multiple comparisons. *p < 0.05, ***p < 0.001, n = 3.** G.** Ctrate synyhase (CS) activity in colon cancer SW480 and DLD1 cell lines treated with vitamin C (5 mM; 6 mM) alone or in combination with NiCl2(0.5 mM). Vitamin C rescues CS inactivation in hypoxic conditions. One-way ANOVA followed by Dunnett's posttest for multiple comparisons. *p < 0.05, n = 3.

As it can be observed (Figure 3A), NiCl_2_ treatment slightly increased PDK-1 expression treatment in DLD1 cells after 6 hours treatment, and Vitamin C (5 mM) strongly inhibited PDK-1 protein expression in both, DLD1 and SW480 CRC cell lines, although this effect was more remarkable in SW480 cell line.

An interesting study carried out by Luo F., *et al* in 2019, reported that not only PDK1 but also Hexokinase II (HKII) that catalyzes the first irreversible step of glycolysis and is often overexpressed in tumor cells, is also able to promote the Warburg effect by phosphorylating alpha subunit of pyruvate dehydrogenase at Serine 293 31. Therefore we wanted to test if the observed vitamin C-dependent PDK1 inhibition also correlated with a reduction in pyruvate dehydrogenase E1-α phosphorylation at Serine 293.

Western blot analysis clearly showed that pyruvate dehydrogenase E1-α phosphorylation at Serine 293 was notably reduced after vitamin C treatment.

Interestingly, Vitamin C treatment did not lead to changes in the expression of pyruvate dehydrogenase in both SW480 DLD1 cancer cells, strongly suggesting that diminished pyruvate dehydrogenase E1-α phosphorylation was driven by the previously observed effect of vitamin C on PDK-1 expression (Figure 3A).

However, these results arised an intriguing query that would call for further research: might also vitamin C treatment affect the expression of Hexokinase II in *KRAS* mutant CRC cells?

The glucose transporter1 (GLUT-1) is another important target of HIF-1, also playing a pivotal role in the Warburg effect. Overexpression of GLUT-1 is often observed in cancer tightly correlating with enhanced cell glycolytic rates and therefore, some interesting attempts have been already carried out in order to target GLUT1 in cancer 32, 33.

Our results show that GLUT1 expression is notably increased in NICL_2_ hypoxic mimicked conditions, but protein presence was also drastically decreased in both cell lines after 6 hour of 5mM vitamin C treatment (Figure 3A).

We wondered whether vitamin C might also affect Hif-1-dependent expression of GLUT-1 and PDK-1. As it is shown (Figure 3B), Real-Time RT-qPCR analysis displayed enhanced mRNA expression of *GLUT-1* and *PDK1* in response to NiCl_2_ but, once again, a notable effect of vitamin C on the cell transcriptional activity was observed. *PDK-1* and *GLUT-1* mRNA were differentially downregulated by vitamin C even in presence of NiCL_2_.

In SW480 cells, *GLUT-1* mRNA was already significantly downregulated after 6h of vitamin C exposure. However, effect of vitamin C on *PDK-1* mRNA expression only could be detected after 12 h of treatment.

Interestingly, a significant effect of vitamin C on PDK-1 and GLUT-1 transcriptional mRNA in DLD1 cells only could be observed after 12 h of vitamin C treatment.

As it was stated above, overexpression of PDK-1 has already been described in CRC 4 and we performed immunohistochemical analysis of PDK1 expression in a number of *KRAS* wild type (n=3) and *KRAS* mutant (G12V) (n=3) tumor biopsies in order to check whether we could detect variability in PDK-1 expression. To our surprise, important differences were observed and remarkably higher presence of PDK-1 was observed in the *KRAS* mutant samples (Figure 3C) suggesting a synergic effect of KRAS mutation and HIF-1 on PDK-1 expression. This interesting result shows a positive correlation between KRAS mutation and PDK1 expression.

In order to check whether vitamin C might modulate *in vivo* tumoral expression of both HIF-1 targets, PDK-1 and GLUT-1, we performed xenograft assays using athymic nude mice Foxn1nu, 7 weeks old (n=10) to observe the putative effect of vitamin C on GLUT1 and PDK1 expression. Tumors from SW480 *KRAS* (G12V) cells were generated and then mice were treated with vehicle or vitamin C (4 gr/kg body weight) for 15 days once daily.

Surprisingly, not only the expression of GLUT-1 but also PDK-1 was heavily reduced in tumors extracted from animals treated with vitamin C, therefore supporting our previous observations with SW480 and DLD1 cell lines (Figure 3D).

PDK-1 and GLUT-1 expression in cancer has previously been associated to an increased stabilization of HIF1α 34, 35, so these results clearly show that vitamin C is interfering with the expression of enzymes that play a pivotal role in the tumoral metabolic shift.

As it has been reported, alterations in the TCA cycle are often observed in hypoxic CRC tumors correlating with HIF-1 expression and *KRAS* mutation, but we observed that vitamin C inhibits (both HIF-1 targets) GLUT-1 and mitochondrial PDK-1 expression in DLD-1 and SW480 CRC cell lines. On the contrary, the overall PDH expression was not affected by vitamin C in both cell lines tested, but we clearly observed modifications in its phosphorylation state, associated with activity, (Figure 3A).

The overall of these data upraise a provocative question: may vitamin C enhance mitochondrial PDH activity, then boosting pyruvate decarboxylation and conversion into acetyl-Coa?

We decided to carry out assays to answer this puzzling question:

The mitochondrial PDH activity and pyruvate decarboxylation are associated to a measurable reduction of NAD to NADH. So, using DLD-1 and SW480 cell extracts, we performed experiments to check putative vitamin C-dependent variations on PDH activity in presence and absence of 500 µM NiCl_2_. Results are depicted in Figure 3E.

In normoxic conditions and absence of NICl_2,_ vitamin C enhanced PDH activity in a significant manner.

As espected, NICL_2_ hypoxic conditions strongly inhibited PDH activity in both *KRAS* mutant cancer cell lines when compared to the control. However, vitamin C enhanced PDH activity even in a higher percentage than that observed for normoxic conditions.

These striking results point out to a novel role of vitamin C refueling the altered mitochondrial TCA cycle with acetyl-Coa and then activating citrate synthase activity in *KRAS* mutant CRC as depicted in Figures 3F and 3G.

These data strongly suggest that if mitochondrial pyruvate decarboxilation and conversion to acetyl-CoA is enhanced by vitamin C, lactate dehydrogenase (LDH) activity and cytoplasmic catalysis of pyruvate to lactate might be inhibited in the same way. Although these promising results point out to an underrated role of vitamin C in the metabolic modulation of cancer cells, some authors have reported that there are some other enzymes of the TCA cycle that show deficient activity or altered expression.

For example, the α-KG dehydrogenase (OGDH) that catalyses the conversion of α-ketoglutarate to succinyl-CoA is downregulated in colorectal cancer as the result of promoter hypermethylation 36.

Isocitrate dehydrogenases (IDHs) are enzymes that catalyze the oxidative decarboxylation of isocitrate. Although rare, mutations of *IDH1* and *IDH2* members have also been observed in colorectal tumors 37. The aberrant functioning of the TCA cycle in colorectal cancer is due to various alterations in enzymes involved in the cycle itself and, probably, other mechanisms yet to be discovered. Therefore, the real potential of vitamin C to modulate the metabolism of tumor cells requires further investigation.

### Vitamin C inhibits ATP generation in SW480 and DLD1 and dissipates the mitochondrial membrane potential (*ΔΨ*m) in SW480 cells

PDK-1 and the pyruvate dehydrogenase complex exert their function inside the mitochondria.

Vitamin C is a water soluble vitamin taken up directly by the cells as l-ascorbic acid (AA) via high affinity/low capacity Na^+^-dependent transporters 1 and 2 (SVCT1 and 2). Cells also intake the vitamin as dehydroascorbic acid (DHA) through hexose transporters, an event followed by the rapid intracellular reduction back to AA 38-40. Vitamin C is then capable to enter the mitochondria via SVCT2 transporter.

Mitochondria are double membrane-enveloped organelles that play a key role in cellular metabolism, redox signaling, calcium homeostasis, and cell fates.

In healthy cells, mitochondria are the main ATP generators, but in many cancer cells, mitochondria do not work properly and energy metabolism shifts from oxidative phosphorylation to active glycolysis, accompanied by an increase in reactive oxygen species generation (ROS) and remarkable increased transmembrane potential (*ΔΨ*m) 41.

In this context, some authors have proposed that mitochondrial pyruvate dehydrogenase kinase (PDK) inhibitors are capable to shift metabolism from glycolysis to glucose oxidation, decreasing *ΔΨ*m in cancer, but not normal, cells 42.

Our previous data strongly pointed out to modulating activities of vitamin C inside the mitochondria, and observed vitamin C-dependent PDK-1 inhibition led us to design assays in order to verify whether vitamin C might also induce changes in the membrane potential in CRC cancer cells.

**Figure 4:**
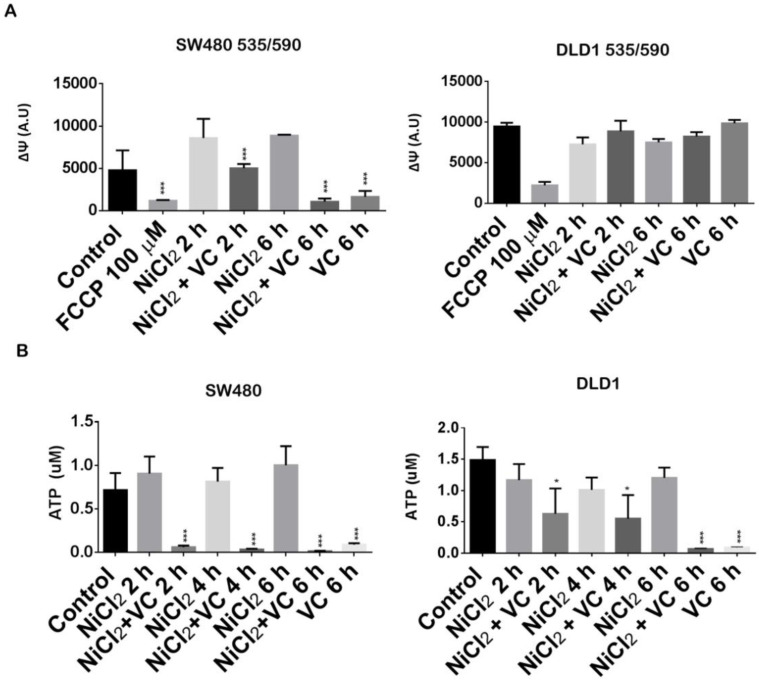
** Vitamin C reduces ATP production and dissipates mitochondrial potential. A.** Mitochondrial membrane potential assay in KRAS mutant DLD1 and SW480 cells treated with vitamin C (5 Mm; 6 mM) and NiCl2 (0.5 Mm) for 2 and 6 h. Vitamin C reduces mitochondrial membrane potential in SW480 cell line. **B.** ATP detection assay in SW480 and DLD1 treated with vitamin C (5 Mm; 6 mM) and NiCl2 (0.5 Mm) for 2, 4 and 6 h. Vitamin C heavily reduces ATP production in the cells. One-way ANOVA followed by Dunnett's posttest for multiple comparisons. *p < 0.05, ***p < 0.001, n = 3.

As it is shown in Figure 4A, mediated-NiCl_2_ hypoxic conditions induced an impontant increase of the mitochondrial transmembrane potential in SW480 cells after 2 and 6 h treatment. Addition of 100 uM of the proton ionophore FCCP (carbonylcyanide p-trifluoromethoxyphenylhydrazone) almost abolished *ΔΨ*m.

Surprisingly, Vitamin C induced a progressive depolarization of the mitochondrial *ΔΨ* and after 6 h of exposure; it was almost completely disrupted in a similar way of FCCP.

Interestingly, different results were obtained for DLD1 cells. In this case, NiCl_2_ hypoxic conditions did not lead to a significant increase of the mitochondrial transmembrane potential (*ΔΨ*m) and Vitamin C was not able to abolish it after 2 and 6 h treatment.

Neither PDK-1 expression nor PDH phosphorylation were observed in DLD-1 cells after 6 h of vitamin C treatment (Figure 3A), but they were both inhibited after 24 h exposure to 5 mM vitamin C. These observations support the increased resistance of DLD-1 cells to vitamin C depicted in Figure 1D.

A possible explanation for the differential activity of vitamin C on SW480 and DLD1 transmembrane potential may rely on the reported differences in the aminoacid sequence of the mitochondrial SVCT2 transporter.

As we previously stated, Vitamin C is taken into the mitochondria in cotransport with Na^+^ via the high affinity/low capacity Na^+^-dependent transporter SVCT2. An interesting work by Mouradov *et al* in 2014, showed that SVCT2 expressed in DLD1 CRC cells display an aminoacid change in position 264 (E264K). Glutamate (or Glutamic acid) is a negatively charged polar aminoacid and it is substituted by a positive Lysine in DLD-1 cells 43. This aminoacid substitution implies changes in the isoelectric point (IP) of SVCT2 that may affect to the kinetics and SVCT2 transport skills.

Interestingly, vitamin C was able to disrupt ATP generation in SW480 and DLD1, although different kinetic was observed for both cell lines (Figure 4B).

Strong inhibition of ATP generation was observed in SW480 in normoxic and hypoxic conditions just after 2 h exposure to 5 mM vitamin C. However, in DLD1 cells we only could report significant inhibition of ATP after 6h treatment of vitamin C treatment, again supporting our previous observations.

## Conclusion

We hereby present compelling data showing a novel and unexpected role of vitamin C in the modulation of altered mitochondrial metabolism in *KRAS*-mutant CRC. In summary, vitamin C induces HIF1α proteasomal degradation, consequently downregulating the expression of its direct targets PDK1 and GLUT1. Downregulated PDK1 correlates with reduced phosphorylation of PDH, implying an elevated enzyme activity. Increased PDH activity involves, then, enhanced pyruvate decarboxylation into acetyl-Coa, strongly pointing out to an important role of vitamin C rewiring the tumor reprogrammed tricarboxylic acid (TCA) cycle. The role of vitamin C in the clinical management of hypoxic *KRAS* mutant colon cancer and importance of normal plasmatic vitamin C (AA) levels in those patients should be reassessed.
