# Supplementary material for: Vitamin C activates pyruvate dehydrogenase (PDH) targeting the mitochondrial tricarboxylic acid (TCA) cycle in hypoxic KRAS mutant colon cancer
Source: Theranostics. 2021 Jan 25;11(8):3595–606. doi: 10.7150/thno.51265 (PMC7914362; doi:10.7150/thno.51265)
Supplement: Supplementary file 1 — Supplementary figures. [file thnov11p3595s1.pdf]

Figure S1. NiCl2 slightly affects cell viability in KRAS mutant colon cancer cells. A. Vitamin C (5 mM), NiCl2 (0.5 mM) and combination dependent apoptosis in KRAS mutant SW480 and DLD1 cell lines. Cells were incubated with respective treatments for 24h and stained with annexin-V-FITC and propidium iodide (PI) for flow cytometry analysis. The populations of apoptotic cells (annexin-V positive/PI negative) and late apoptotic cells (annexin positive/PI positive) were evaluated as a percent of total cells. Nicl2 alone slightly induces apoptosis while vitamin C activates this process massively. **B.** Cell viability analysis of SW480 and DLD1 cell lines treated with incremental vitamin C concentrations (1,3,5 mM) for 24h in combination with Nicl2. Nicl2 alone hardly affects cell viability while ascorbate exposure is extremely cyitotoxic. **C** Time dependent cell viability assay in SW480 and DLD1 cell lines incubated with NiCl2 alone or in combination with vitamin C for 4, 8, 12 and 24 hr. Cell viability is not affected significantly after 24h Nicl2 exposure. One-way ANOVA followed by Dunnett's posttest for multiple comparisons. *p < 0.05, ***p < 0.001, n = 3.

Figure S2. MG132 does not affect neither KRAS mutant colon cancer cells viability nor interferes with vitamin C activity. Cell viability analysis of SW480 and DLD1 cell lines treated with MG 132 (25 μM) and vitamin C (5 mM; 6 mM) for 1, 3 and 6 hr. MG 132 does not interfere with the loss of viability induced by vitamin C. One-way ANOVA followed by Dunnett's posttest for multiple comparisons. *p < 0.05, ***p < 0.001, n = 3.

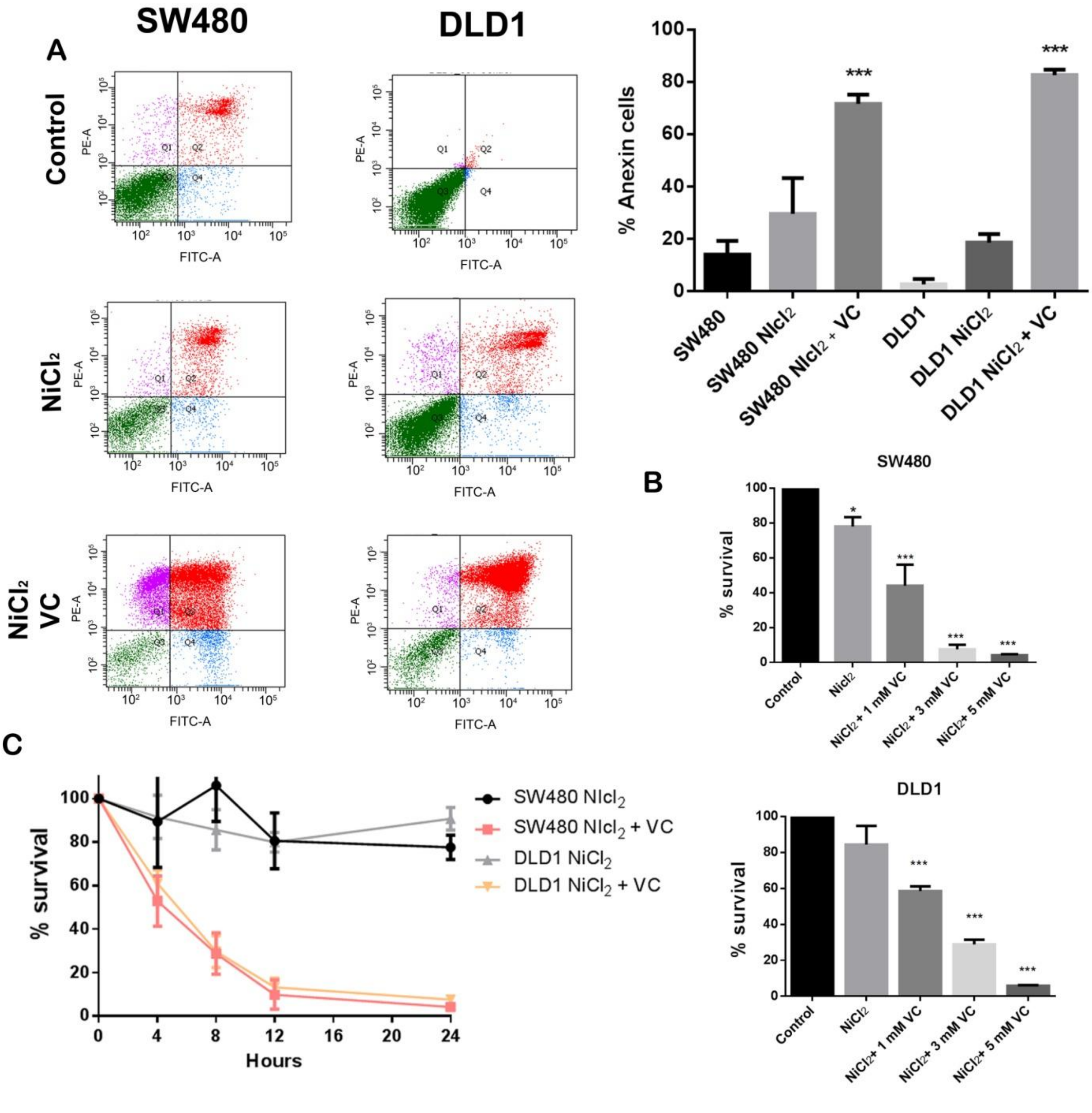
A
SW480
DLD1
Control
NiCl₂
NiCl₂ VC
PE-A
FITC-A
Q1
Q2
Q3
Q4
% Anexin cells
***
SW480
SW480 NIcl2
SW480 NIcl2 + VC
DLD1
DLD1 NiCl2
DLD1 NiCl2 + VC
B
SW480
% survival
*
Control
NIcl2
NiCl2 + 1 mM VC
NiCl2 + 3 mM VC
NiCl2+ 5 mM VC
DLD1
NiCl2
NiCl2+ 1 mM VC
NiCl2+ 3 mM VC
NiCl2+ 5 mM VC
C
SW480 NIcl2
SW480 NIcl2 + VC
DLD1 NiCl2
DLD1 NiCl2 + VC
Hours

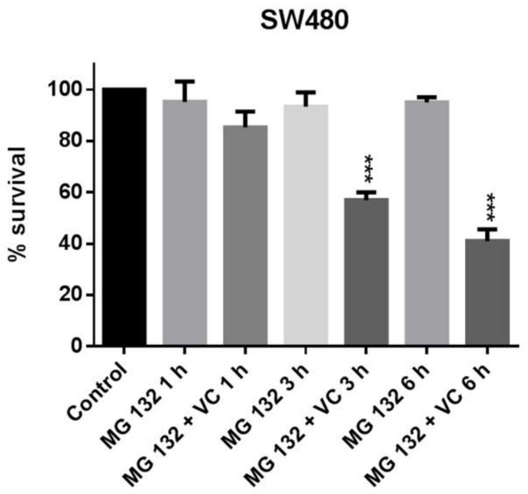
SW480
% survival
0
20
40
60
80
100
***
***
Control
MG 132 1 h
MG 132 + VC 1 h
MG 132 3 h
MG 132 + VC 3 h
MG 132 6 h
MG 132 + VC 6 h

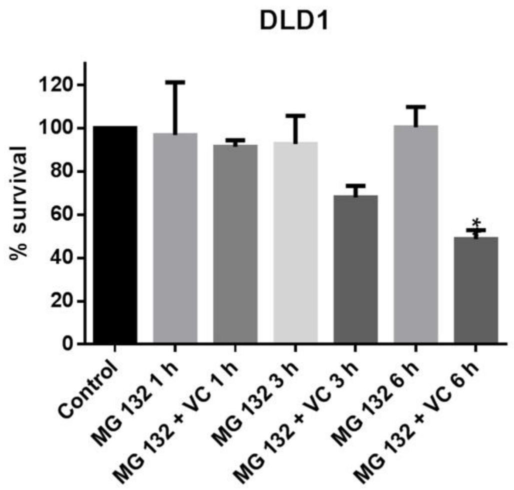
DLD1
% survival
0
20
40
60
80
100
120
*
Control
MG 132 1 h
MG 132 + VC 1 h
MG 132 3 h
MG 132 + VC 3 h
MG 132 6 h
MG 132 + VC 6 h
